# Global mental health should engage with the ethics of involuntary admission

**DOI:** 10.1186/s13033-021-00448-0

**Published:** 2021-03-02

**Authors:** Marisha N. Wickremsinhe

**Affiliations:** Ethox Centre and Wellcome Centre for Ethics and Humanities, Nuffield Department of Population Health, University of Oxford, Oxford, UK

**Keywords:** Involuntary admission, Global mental health, CRPD

## Abstract

Global mental health, as a field, has focused on both increasing access to mental health services and promoting human rights. Amidst many successes in engaging with and addressing various human rights violations affecting individuals living with psychosocial disabilities, one human rights challenge remains under-discussed: involuntary inpatient admission for psychiatric care. Global mental health ought to engage proactively with the debate on the ethics of involuntary admission and work to develop a clear position, for three reasons. Firstly, the field promotes models of mental healthcare that are likely to include involuntary admission. Secondly, the field aligns much of its human rights framework with the UN Convention on the Rights of Persons with Disabilities, which opposes the discriminatory use of involuntary admission on the basis of psychosocial disability or impairment. Finally, global mental health, as a field, is uniquely positioned to offer novel contributions to this long-standing debate in clinical ethics by collecting data and conducting analyses across settings. Global mental health should take up involuntary admission as a priority area of engagement, applying its own orientation toward research and advocacy in order to explore the dimensions of when, if ever, involuntary admission may be permissible. Such work stands to offer meaningful contributions to the challenge of involuntary admission.

## Introduction

The field of global mental health roots its development in interdisciplinary research across multiple domains—including anthropology, medicine and genetics, epidemiology, psychology, sociology, advocacy—with the aim of promoting “mental health for all” [1]. Global mental health has made significant strides not only in highlighting the prevalence of mental ill-health, but also in developing, piloting, and promoting paths toward increasing access to mental healthcare services, especially through primary care and in settings without specialist practitioners [1]. These achievements required massive research efforts, especially in implementation science, as well as collaborative advocacy on the part of service users, academics, governments, and international funding and agenda-setting bodies. Global mental health’s driving force has been motivated, in large part, by appeals to parity between mental and physical health, by proof of cost-effectiveness for treating mental disorders (and, inversely, cost burdens of untreated mental disorders), and critically, by the adoption of a human rights framework for mental health, recently characterized as “mental health as a fundamental human right” [1].

Global mental health’s adoption of a human rights framework is bolstered by principles of the UN Convention on the Rights of Persons with Disabilities (CRPD). The CRPD enshrines a range of human rights protections for individuals living with disabilities, including psychosocial disabilities; one critical human rights protection afforded by the CRPD is freedom from involuntary admission on the basis of impairment. Yet, engagement with the ethics of involuntary admission—a long-standing and widely debated human rights challenge in psychiatry—has been surprisingly absent from discussion in the field of global mental health.

## Limited engagement with involuntary admission in global mental health

Involuntary admission received little attention in seminal agenda-setting papers in global mental health, among them the 2007 and 2011 *Lancet* series on global mental health:

- The 2007 *Lancet* series on global mental health included “involuntary admission as a proportion of all admissions” as a secondary indicator to “ensure least restrictive practice” in its final call to action paper [2].
- The 2011 *Lancet* series on global mental health included a paper on human rights violations, but mentioned involuntary treatment only twice, both times in the context of the right to appeal or contest detention (as opposed to challenging the legal criteria used to justify the practice), and included only a brief discussion of arbitrary detention as occurring in many instances ‘unlawfully’ (again without explicitly engaging with the ethics of the practice itself) [3].

Global mental health’s priorities and agenda have been refreshed by the 2018 *Lancet* Commission on global mental health and sustainable development, which sought to align global mental health with the 2015 Sustainable Development Goals more broadly. The Commission also charted a course for the field’s future progress, considering not only the global mental health field’s own achievements to date, but also broader shifts in the landscape of global health and human rights. The 2018 *Lancet* Commission, in centering a human rights framework, does not take a clear position on the ethics of involuntary admission. Instead, the authors reproduce existing debates about the practice, discussing the mandate of the CRPD—to eliminate the use of involuntary admission—alongside critics’ counterarguments, for example, that prohibition of involuntary admission under any circumstance could “inadvertently undermine the right to health”,^1^ and even the rights to freedom and justice [1]. Ultimately, the 2018 *Lancet* Commission does not take a stand on the ethics of involuntary admission, nor does the Commission suggest that excavating this key human rights issue in psychiatric practice should, itself, be a critical component of global mental health’s research agenda.

Some may contend that the lack of attention to involuntary admission in the global mental health agenda has a simple explanation: involuntary admission just *isn’t* a priority for the field of global mental health, especially considering the other grave human rights violations faced by individuals living with psychosocial disabilities. In fact, this very point is implicit in the arguments advanced in global mental health. When human rights violations are discussed broadly, lack of access to services is centered, which then positions “increasing access to services”, or “scaling up”, as the mechanism for *promoting* human rights for individuals living with mental disorders [3]. In advocating for increased access to services, however, the human rights violations that too often take place *within* services are seldom acknowledged. Moreover, much work in global mental health adopts an implicit assumption that increasing access to services, especially community-based services, will necessarily decrease—if not eliminate—the need for inpatient services [4], and, thus, for involuntary admissions. The tacit argument is that global mental health can simply avoid engaging with questions about involuntary admission by vastly scaling up primary care and community-based services.

But can we feasibly scale up primary care and community-based services to such a degree that the question of involuntary admission is off the table? The need for inpatient care, even when other services are sufficiently scaled up, is widely accepted; countries with robust community mental healthcare retain the need for complementary inpatient services [5]. Though few would argue that we can do away with inpatient services entirely, opponents may offer the following counterargument: global mental health need not address involuntary admission because, even if inpatient services are a mainstay of mental healthcare, these services are not ‘accessed’ involuntarily. However, available data suggests that a significant proportion, if not a majority, of psychiatric hospital admissions are indeed involuntary, regardless of country income status [6]. Accepting that involuntary admission is likely to remain part of the model of mental healthcare service provision that global mental health seeks to promote, then, I suggest that the field has a responsibility to engage with, and ultimately take a stance on, the ethics of involuntary admission.

## Global mental health’s responsibility to engage with involuntary admission

Some might suggest that the ethics of involuntary admission, though relevant to global mental health, is not one of importance for the field to directly address itself, as the ethics of involuntary admission falls outside global mental health’s realm. The permissibility of involuntary admission remains a live debate in the clinical ethics literature—a debate renewed by the CRPD; against this backdrop of wide disagreement, then, we might think it reasonable for global mental health *not* to take a stand. However, even if taking a position on the permissibility of involuntary admission under specific criteria, if any, falls outside global mental health’s scope, I contend that engaging with involuntary admission, and conducting relevant research on rates, perspectives, and practices—ultimately in service of deriving a position on its permissibility—ought to be a priority for global mental health as a field for three reasons.

Firstly, the dominant narrative of global mental health promotes particular models of mental healthcare that include inpatient services [7]. By failing to address involuntary admission, then, the field runs the risk of exporting a model of service provision (largely modeled after Western systems) that itself perpetuates human rights violations [8], or that simply “[transfer] persons from one form of coercion to another” [9]. Global mental health must engage meaningfully with the ethical arguments concerning, and the legal regulation of, involuntary admission if the field intends to coherently integrate its priority of increasing access to services within a human rights framework. That is, as long as global mental health continues to advocate for the availability of inpatient services within mental health systems, even if only as a small component of these systems, the field must contend with the reality of how inpatient services are often ‘accessed’. Given that the global mental health agenda promotes a model of mental healthcare that leaves open the possibility for at least some, if not a significant number of, involuntary admissions (as available data suggests), the field has a responsibility to meaningfully address—or at least contribute to debate on—the ethics of the practice.

Secondly, global mental health must ensure that its human rights framework, in aligning with the CRPD, takes seriously the full implications of the treaty, including in applying the specific interpretative requirements of the Convention. Even if global mental health does not take an abolitionist stance against involuntary admission across the board, the field’s continued lack of engagement with the topic risks a piecemeal approach to human rights. At present, the field appears to pick and choose elements of the CRPD’s mandates as is helpful to its aims, instead of fully engaging with the treaty’s principles. Of course, people living with psychosocial disabilities face a range of human rights violations, and some in the field may argue that violations related to involuntary admissions are secondary to other abuses. Nevertheless, commitment to a human rights framework requires commensurate attention to the kinds of human rights violations that may accompany increased ‘access’ to services. While important strides have been made at a global scale to address human rights within the context of hospital admissions (e.g. the World Health Organization’s QualityRights Initiative), the field needs to directly engage with the debate—interrogating central ethical tensions arising in the use (or non-use) of involuntary admission, and taking a clear position to ultimately chart a course for the role, if any, of involuntary admission in mental healthcare service provision.

Finally, involuntary admission ought to be a priority for global mental health because the field’s research orientation is uniquely positioned to meaningfully contribute to the debate on the ethics of the practice. That is, beyond global mental health’s clear responsibility to address involuntary admission, as outlined, research on involuntary admission that adopts a global mental health lens stands to offer useful insights: illuminating best practices, exploring cultural and contextual nuances, and expanding the range of stakeholder perspectives surveyed. Critical areas of research include implementation studies of recently reformed mental health legislation, epidemiological studies of rates and correlates of involuntary admission, and qualitative studies eliciting service user perspectives. Drawing on both the field’s interdisciplinarity as well as its focus on adapting research findings across contexts, global mental health should pursue research in these core areas. Such research stands to offer not only diverse understandings of the ethical tensions arising in the use of involuntary admission in different contexts, but also the opportunity to chart a path forward for addressing the ethics of involuntary admission as a field. Counter to arguments that addressing the ethics of involuntary admission falls outside of global mental health’s domain, I suggest that the field could make important and distinctive contributions to this debate, especially against the backdrop of growing calls for research on involuntary admission globally [10].

## Conclusions

Global mental health’s agenda has inadequately addressed the ethics of involuntary admission, even while aligning its human rights framework with the CRPD, which strongly opposes involuntary admission for individuals living with psychosocial disabilities. The field should engage with the ethics of involuntary admission—interrogating core claims in the debate with the ultimate aim of taking a clear position—for three reasons. Firstly, global mental health promotes a model of service provision that inevitably includes involuntary admission, and therefore has a responsibility to engage with the debate on the ethics of the practice. Secondly, because global mental health’s human right framework draws heavily on the CRPD, the field’s continued lack of engagement on involuntary admission risks a piecemeal approach to human rights. Finally, global mental health is uniquely positioned to offer novel insights into practices and perspectives related to involuntary admission by applying its own orientation toward research to explore various dimensions of involuntary admission and, ultimately, contribute meaningfully to the debate.

## Data Availability

Not applicable.

## Abbreviation

CRPD: Convention on the Rights of Persons with Disabilities.

## Footnotes

1 The “right to health”, shorthand for the “right to the enjoyment of the highest attainable standard of physical and mental health”, does, by definition, include freedom *from* non-consensual medical care, including freedom from involuntary admission, according to the Office of the United Nations High Commissioner for Human Rights (Human Rights Factsheet No. 31, 2008).
